# Hypoglycemia in Non-Diabetic In-Patients: Clinical or Criminal?

**DOI:** 10.1371/journal.pone.0040384

**Published:** 2012-07-02

**Authors:** Krishnarajah Nirantharakumar, Tom Marshall, James Hodson, Parth Narendran, Jon Deeks, Jamie J. Coleman, Robin E. Ferner

**Affiliations:** 1 Public Health, Epidemiology and Biostatistics, University of Birmingham, Birmingham, United Kingdom; 2 ePrescribing Research team, University Hospital Birmingham, Birmingham, United Kingdom; 3 Department of Diabetes, University Hospital Birmingham, Birmingham, United Kingdom; 4 School of Clinical and Experimental Medicine, University of Birmingham, Birmingham, United Kingdom; 5 West Midlands Centre for Adverse Drug Reactions, City Hospital, Birmingham, United Kingdom; Universita Magna-Graecia di Catanzaro, Italy

## Abstract

**Background and Aim:**

We wished to establish the frequency of unexpected hypoglycemia observed in non diabetic patients outside the intensive care unit and to determine if they have a plausible clinical explanation.

**Methods:**

We analysed data for 2010 from three distinct sources to identify non diabetic hypoglycaemic patients: bedside and laboratory blood glucose measurements; medication records for those treatments (high-strength glucose solution and glucagon) commonly given to reverse hypoglycemia; and diagnostic codes for hypoglycemia. We excluded from the denominator admissions of patients with a diagnosis of diabetes or prescribed diabetic medication. Case notes of patients identified were reviewed. We used capture-recapture methods to establish the likely frequency of hypoglycemia in non-diabetic in-patients outside intensive care unit at different cut-off points for hypoglycemia. We also recorded co-morbidities that might have given rise to hypoglycemia.

**Results:**

Among the 37,898 admissions, the triggers identified 71 hypoglycaemic episodes at a cut-off of 3.3 mmol/l. Estimated frequency at 3.3 mmol/l was 50(CI 33–93), at 3.0 mmol/l, 36(CI 24–64), at 2.7 mmol/l, 13(CI 11–19), at 2.5 mmol/l, 11(CI 9–15) and at 2.2 mmol/l, 8(CI 7–11) per 10,000 admissions. Admissions of patients aged above 65 years were approximately 50% more likely to have an episode of hypoglycemia. Most were associated with important co-morbidities.

**Conclusion:**

Significant non-diabetic hypoglycemia in hospital in–patients (at or below 2.7 mmol/l) outside critical care is rare. It is sufficiently rare for occurrences to merit case-note review and diagnostic blood tests, unless an obvious explanation is found.

## Introduction

Hypoglycemia in patients without diabetes has many potential causes [1]–[13], one of which is the malicious administration of insulin [14]–[19]. Murder may be under-reported worldwide [19]. In reported cases, perpetrators are often carers or clinical staff, and victims their patients [19]. Prominent cases in the United Kingdom have involved multiple deaths of elderly hospital patients [15], and of children [17], [18]. Similar cases have occurred in the United States [20]–[22], at a Vienna medical centre [23], and at old-age homes in Belgium and the Netherlands [19], [20]. While confirmation of insulin poisoning requires serum insulin and C-peptide concentrations, the first suspicion may be raised by the occurrence of unexplained hypoglycemia [14]. Better knowledge of the frequency of hypoglycemia in hospital patients is required to understand these complex forensic and clinical questions.

Hypoglycemia is common in diabetic in-patients in any setting [24]–[26]. It is also common in non-diabetic patients in critical care setting [27], [28], partly because of attempts to achieve tight blood glucose control, although this has now been shown to be harmful [28], [29]. Few studies [1], [4], [11], [30], [31] have examined the incidence of hypoglycemia in non-diabetic patients (‘non-diabetic hypoglycemia’) outside the critical care setting, although this is potentially important both clinically and forensically. Previous reports of the frequency of non-diabetic hypoglycemia have included small numbers of patients [11], [31] or have included diabetic as well as non-diabetic patients [1], [30], [31] and all [1], [4], [11], [31] but one [30] included only elderly patients.

We wished to establish the frequency of unexpected hypoglycemia observed in non diabetic patients outside the intensive care unit in University Hospital Birmingham (UHB), a large acute hospital with approximately 1200 beds. The hospital has a purpose-designed computer-based patient information system, the Patient Information and Communication System (PICS), which records laboratory results, electronic observations and medication orders, and a Patient Administration System (PAS) which records discharge diagnostic codes. We therefore had the opportunity to analyse retrospective data available for the year 2010 from three distinct sources: blood glucose concentration measurements, both from the bedside and the laboratory; medication records for treatments (glucose, glucagon) commonly given to reverse hypoglycemia; and diagnostic codes for individual patients. Each data source identifies a different sample of all hypoglycemic episodes, but no single data source can be regarded as definitive. We therefore used capture-recapture methods to establish the likely true rate of hypoglycemia in non-diabetic in-patients outside intensive care units, using data from all three sources.

## Methods

### Data sources

We identified all patients 16 years old and above who were logged in PAS during the calendar year 2010 as hospital admissions, whether elective or non-elective. PAS data were linked to PICS data and patients with a recorded diagnosis of diabetes in the PAS or who were identified in PICS as having received treatment with anti-diabetic medication were excluded. This broad exclusion criterion was used because our main purpose was to determine the frequency of hypoglycemia that could not be explained by the use of prescribed hypoglycemic agents. We included patients admitted to the intensive care unit (ICU) in the denominator, as they invariably have a period of stay outside ICU (susceptible population). This identified a population of non-diabetic in-patients who could suffer a hypoglycemic episode outside the critical care setting.

We identified episodes of hypoglycemia in three ways. We directly identified low blood glucose concentrations from the PICS database, which captured both bedside and laboratory blood glucose estimations; we indirectly identified hypoglycemia from prescribed treatments for hypoglycemia whose administration was recorded in the PICS database; and we identified diagnostic codes for hypoglycemia from the PAS database. We regarded any one of these as a trigger event that signalled potential hypoglycemia. If a trigger occurred during a period of time the patient spent in ICU the patient's case was excluded from the numerator.

### Cut-off value for hypoglycemia

Various blood glucose concentrations have been used to define hypoglycemia in non-diabetic patients. Previous studies have used 2.7 [4], [30], 3.0 [31] and 3.3 mmol/l [1], [11]. Meanwhile 2.2 mmol/l is used to define severe hypoglycemia in diabetic patients [26] and 2.5 mmol/l has been used for forensic investigations [32]. Considering the uncertainty, we analysed the data at different values of blood glucose concentration, to establish the effect on perceived occurrence. We did not differentiate between laboratory blood glucose values and point-of-care blood glucose values, or consider the type of equipment used to measure glucose values.

### Medication as an indirect indicator of hypoglycemia

We examined electronic prescription records for medication used to treat severe hypoglycemia, to establish whether these may serve as triggers in detecting hypoglycemia in non-diabetic patients. The triggers extracted from PICS were intramuscular glucagon injection, intravenous glucose 10%, 20%, and 50% solutions; and oral glucose 40% gel. The case-notes of patients who received any of these but had neither a prescription for anti-diabetic medication in PICS nor a diagnostic code for diabetes in PAS were reviewed to establish whether they had in fact been hypoglycemic. Reasons for false positives were documented.

### Discharge diagnostic codes for hypoglycemia as an indirect indicator

We examined ICD10 discharge diagnostic codes for hypoglycemia (E15, E16.0, E16.1, and E16.2) in the PAS system for the year 2010. The case-notes of patients with these ICD 10 codes who had neither a prescription for anti-diabetic medication in PICS nor a diagnostic code for diabetes in PAS were reviewed to determine the validity of the triggers in identifying non-diabetic hypoglycemia. Again reasons for false positives were recorded.

### Electronic point-of-care and laboratory blood glucose concentrations as direct indicators of hypoglycemia

From available electronic observations derived from PICS, point-of-care or laboratory blood glucose values were used to identify patients who had been hypoglycemic during their in-patient spell. We reviewed the case-notes of patients categorised as non-diabetic by the criteria described above.

### Determining the causes of hypoglycemia in non-diabetic patients

Information on diagnosis that was noted in the discharge diagnostic codes from PAS was first verified as an accurate description by case-note review. Any missed diagnostic codes were documented. A checklist of potential causes derived from previous literature was used to identify possible reasons for the hypoglycemia [1]–[13]. Because ICD 10 codes are limited in their ability to describe aetiology, for every patient with a blood glucose value less than or equal to 2.7 mmol/l, two investigators (KN and PN) made a judgment, based on the co-morbidity and patient condition, as to whether the patient may have had an unexplained hypoglycemia.

### Statistical analysis

Data were analysed using STATA® 10 software [33]. Cases identified by any of the three triggers were used to estimate the incidence of hypoglycemia in non-diabetic patients. As none of the three data sources is complete we used the capture-recapture technique for three sources [34]. Capture- recapture methods have been used in health care to estimate population prevalence using multiple incomplete sources [35]–[40]. In summary, eight log-linear models, each specifying different interactions between the three sources, are derived to estimate the size of the total population. The layout of the three-source models is given in supporting information (Text S1). Calculations are based on the overlap between the three sources. The best estimate of the eight given is chosen using the Akaike information criteria (AIC) and the Bayesian information criteria (BIC). The 95% confidence intervals around the estimates were calculated using the goodness-of-fit based method [41]. An example of the working using the three source model is given in supporting information (Text S2) [42]. We estimated the frequency at different cut-off values: 3.3, 3.0, 2.7, 2.5 and 2.2 mmol/l. Frequency is reported as a count for the observed number of admissions of patients who did not have diabetes or receive diabetic medication in a non critical care setting for the year 2010. We repeated the same analysis stratifying the population by age into those 65 years and above and those who were less than 65 years old.

The study was considered by the Clinical Governance Support Unit of the University Hospital Birmingham (which has the function of an institutional review board to determine whether a study requires ethics approval and patient consent or is considered to be audit of clinical practice). The Unit approved the study as an audit that used routinely collected data for service improvement, involved no experiment, and did not require written patient consent.

## Results

**Figure 1:**
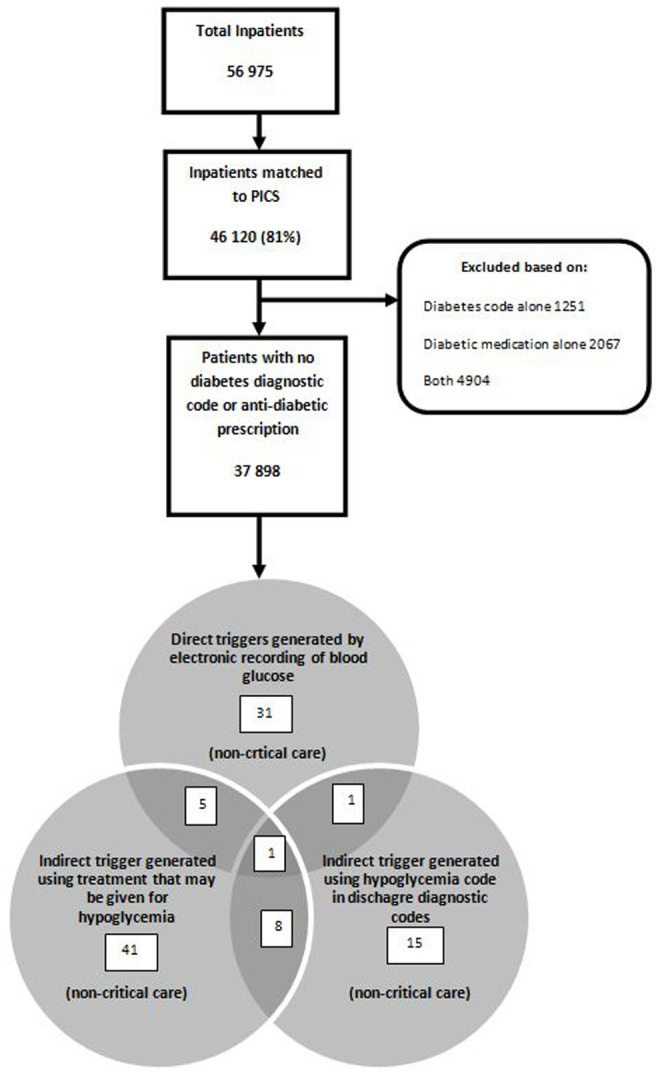
Flow diagram of non diabetic hypoglycaemia triggers generated.

There were 56 975 inpatient admissions to the hospital in 2010. We were able to match 81% of the PAS data to the PICS system (46 210 admissions). Among them 37 898 were categorised as non-diabetic based on the absence of either discharge diagnostic code of diabetes or a record of the prescription of diabetic medication. There were 38 direct triggers from the blood glucose concentrations using 3.3 mmol/l as the cut-off value, 55 indirect triggers using treatment for hypoglycemia and 25 indirect triggers using discharge diagnostic codes, yielding a total of 102 non-diabetic admissions with at least one episode of hypoglycemia, excluding overlaps between the three sources (Figure 1). Case-notes were available for review for 95 (93%) admissions.

### The frequency of hypoglycemia among non-diabetic patients in non critical care setting

**Figure 2:**
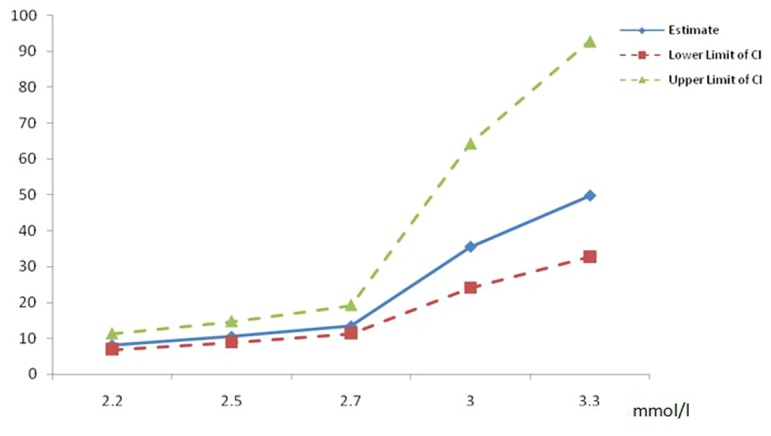
Number of hypoglycaemia episodes –v- threshold blood glucose concentration (mmol/l) and upper and lower 95% confidence bounds per 10,000 admissions.

In combination the triggers identified 71 unique admissions with hypoglycemic episodes at a cut-off of 3.3 mmol/l, 59 at 3 mmol/l, 37 at 2.7 mmol/l, 30 at 2.5 mmol/l and 23 at 2.2 mmol/l. Each of these admissions was of a unique patient. Using the capture-recapture method at 3.3 mmol/l cut-off, an estimate of 189 (95%CI 124 to 352) hypoglycemic episodes is predicted in a non diabetic population of 37 898, giving a cumulative incidence of 50 per 10 000 admissions (95%CI 33–93). Estimated cumulative incidence at 3.0 mmol/l was 36 (95%CI 24–64), at 2.7 mmol/l, 13 (95%CI 11–19), at 2.5 mmol/l, 11 (95%CI 9–15) and at 2.2 mmol/l, 8 (95%CI 7–11) per 10 000 admissions (Figure 2).

Analysis showed admissions with an age above 65 years were more (approximately 50% more) likely to have an episode of hypoglycemia compared to the younger age group at all cut-off points. Estimated frequency above the age of 65 years at 3.3 mmol/l was 55 (95%CI 32–149), at 3.0 mmol/l, 39 (95%CI 24–158), at 2.7 mmol/l, 18 (95%CI 15–27), at 2.5 mmol/l, 15 (95%CI 13–23) and at 2.2 mmol/l, 13 (95%CI 11–21) per 10 000 admissions (Table S1).

### Causes of non-diabetic hypoglycemia

Characteristics of the non-diabetic patients who had hypoglycemia at a cut-off point of 3.3 and 2.7 mmol/l are given in Table 1. Most patients (>90%) were admitted as an emergency. The commonest co-morbidities linked to hypoglycemia were sepsis, kidney disease, and alcohol dependence. Others included pneumonia, liver disease, cancer, and self-harm with hypoglycemic agents. Most patients had multiple possible reasons for their hypoglycemia (Table S2). Detailed case-note review of those with blood glucose concentrations less than 2.7 mmol/l revealed seven patients who did not have a plausible reason to explain the occurrence of hypoglycemia. However all seven were either admitted for investigation of hypoglycemia that occurred elsewhere or had an episode that was noted on admission; there was no unexplained hypoglycemia that occurred after admission during inpatient stay (Figure S1). Over 30% of patients whose blood glucose concentration was lower than 3.3 mmol/l, and nearly 40% of those whose blood glucose concentration fell below 2.7 mmol/l, died.

**Table 1:** Characteristics of the patients identified as non diabetic hypoglycemic patients.

Patient Characteristics	Glucose <3.3 mmol/l (N = 71)	Glucose <2.7 mmol/l (N = 37)
Age mean (SD) years	**59.2 (22.5)**	**60.2 (23.6)**
Age Group		
<65 years	**41 (57.7)**	**20 (54.1)**
>65 years	**30 (42.3)**	**17 (45.9)**
Gender N (%)		
Male	**37 (52.1)**	**20 (54.1)**
Female	**34 (47.9)**	**17 (45.9)**
Ethnicity N (%)		
White	**50 (70.4)**	**26 (70.3)**
Asian	**8 (11.3)**	**4 (10.8)**
Black	**5 (7.0)**	**3 (8.1)**
Other	**8 (11.3)**	**4 (10.8)**
*Social class N (%)		
Least deprived 1	**4 (5.6)**	**0 (0.0)**
2	**7 (9.9)**	**3 (8.1)**
3	**17 (23.9)**	**10 (27.0)**
4	**11 (15.5)**	**5 (13.5)**
Most deprived 5	**31 (43.7)**	**18 (48.6)**
Type of Admission N (%)		
Elective	**7 (9.9)**	**2 (5.4)**
Emergency	**64 (90.1)**	**35 (94.6)**
In-patient death N (%)		
Yes	**24 (33.8)**	**14 (37.8)**
No	**47 (66.2)**	**23 (62.2)**
Length of stay median (IQR) days	**6.92 (11.54)**	**7.42 (13.88)**
On admission %	**22 (31.0)**	**17 (45.9)**
#Aetiology for hypoglycaemia		
Sepsis	**20 (28.2)**	**11 (29.7)**
Renal Disease	**20 (28.2)**	**12 (32.4)**
Alcohol	**15 (21.1)**	**11 (29.7)**
Pneumonia	**17 (23.9)**	**6 (16.2)**
Liver disease	**9 (12.7)**	**6 (16.2)**
Congestive Heart Failure	**9 (12.7)**	**6 (16.2)**
Cancer	**10 (14.1)**	**2 (5.4)**
Self harm	**4 (5.6)**	**4 (10.8)**
Under investigation for hypo occurring elsewhere	**5 (7.0)**	**3 (8.1)**

* Social class based on income deprivation score. Adds up to 70 & 36 instead of 71 & 37 respectively at 3.3 and 2.7 mmol/l due to one missing post code.

# Will add up to more than 100% due to multiple co-morbidities in patients. All co-morbidities are based on ICD 10 code (verified by case-note review). Where ICD 10 code was not available a documentation of the diagnosis in case-note was accepted.

## Discussion

### Summary of findings

Non-diabetic hypoglycemia is rare in hospital in-patients. We estimate that, with a cut-off value of 2.7 mmol/l, 13 (95% CI 11–19) episodes per 10 000 admissions occurred in one year; and with a cut-off value of 3.3 mmol/l 50 (95% CI 33–93) episodes per 10 000 admissions per year. Estimates are higher in patients above the age of 65 years (39 and 55 per 10 000 admission respectively at cut-off values of 2.7 and 3.3 mmol/l). All the cases of hypoglycemia that occurred after admission could be explained by co-morbid conditions, principally alcohol dependence, renal failure, and sepsis.

### Comparison with other studies

Our estimates are similar to previous studies [4], [30], [31] except that of Mannucci et al [11] who reported an incidence of 8.6% in a small (678 patients) elderly population from a single medical unit. However the study was mainly based on routine blood glucose concentrations measured in the fasting state, defined hypoglycemia at a cut-off point of 3.3 mmol/l, and examined patients with a mean age of 81 years admitted with co-morbidities commonly associated with hypoglycemia.

Our study supports the previous observations that non-diabetic patients who develop hypoglycemia are more likely to die than those who do not [1], [11], [30]. Similarly, hypoglycemia in our study was often associated with renal disease, sepsis or pneumonia, and alcohol dependence, and other co-morbid diseases, in accord with previously suggested associations [8], [11], [13], [30]. Hypoglycemia can be a marker of poor prognosis, so that identified patients may need intense monitoring and management. If a cause for severe spontaneous hypoglycemia is not obvious, then the correct samples should be taken prior to treatment so that a diagnosis can be made. The samples can subsequently be analyzed for insulin, C-peptide, and other markers along with confirmatory laboratory glucose measurement and routine blood samples for renal disease, liver disease, and sepsis.

### Limitations

Our study was retrospective. All three sources of information were incomplete, as we expected. We were able to overcome this by using the capture-recapture technique, and provide estimates of the true means with relatively narrow confidence intervals. Our study involves only one large hospital in UK. The hospital has specialist renal and liver units and electronic recording of near-patient blood glucose tests, which will have increased the observed frequency of hypoglycaemia. However, we accept that observed rates may be different elsewhere, and depend at least in part on the patient population and on the nature and frequency of testing.

### Conclusion

Significant non-diabetic hypoglycemia in hospital in–patients (at or below 2.7 mmol/l) outside critical care is rare. It is sufficiently rare for occurrences to merit case-note review and diagnostic blood tests, unless an obvious explanation is found.

#### Disclaimer

The views expressed in this publication are not necessarily those of the NIHR (Funder), the Department of Health, NHS Partner Trusts, University of Birmingham or the CLAHRC-BBC Theme 9 Management/Steering Group.

## Supporting Information

Figure S1 **Plausible explanation for hypoglycaemia.**
(DOCX)

Table S1 **Age based best estimates for non diabetic hypoglycemia in hospitalised patients in non critical care setting.**
(DOCX)

Table S2 **Matrix showing co-morbidity linked to hypoglycaemia in patients with glucose <2.7 mmol/l (N = 37).** All diagnosis are based on the ICD 10 coding and mostly reflect the codes used in Charlson co-morbidity (Except self harm, dialysis, sepsis and Pneumonia).
(DOCX)

Text S1 **Three source model layout for estimating the numbers of non-diabetic hypoglycaemia.**
(DOCX)

Text S2 **Example: Three source model for estimating the numbers of non diabetic hypoglycaemia (<3.3 mmol/l).**
(DOCX)
