# Supplementary material for: Hypoglycemia in Non-Diabetic In-Patients: Clinical or Criminal?
Source: PLoS One. 2012 Jul 2;7(7):e40384. doi: 10.1371/journal.pone.0040384 (PMC3388042; doi:10.1371/journal.pone.0040384)
Supplement: Figure S1 — Plausible explanation for hypoglycaemia. (DOCX) [file pone.0040384.s001.docx]

## Figure S1: Plausible explanation for hypoglycaemia


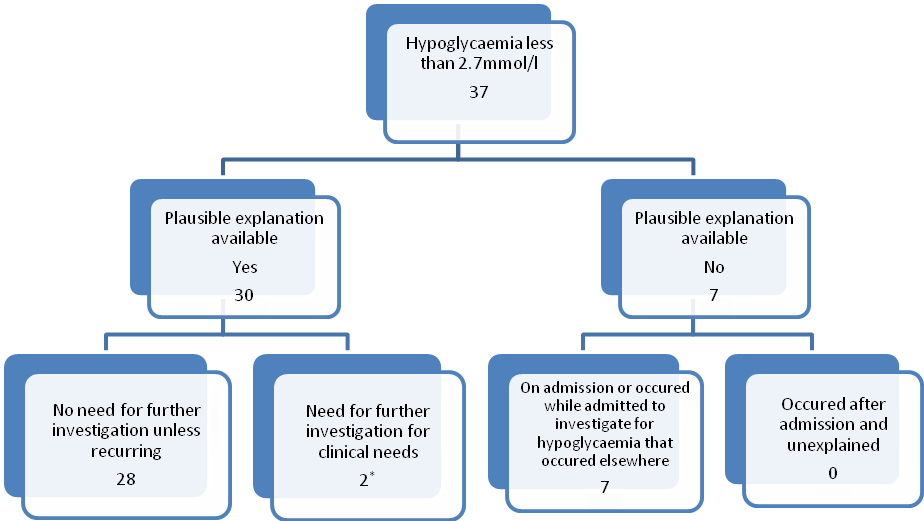


*****One patient had leiomyosarcoma and hypoglycaemia. The association between these have been reported in association with insulin-like growth factor 1, which had not been determined in this patient.

One patient had SLE and admitted with sepsis but was very young (23 years) and had a blood glucose concentration less than 1.5 mmol/l. While hypoglycaemia may have been linked to sepsis, other clinical explanations had not been excluded.
