# Supplementary material for: Hypoglycemia in Non-Diabetic In-Patients: Clinical or Criminal?
Source: PLoS One. 2012 Jul 2;7(7):e40384. doi: 10.1371/journal.pone.0040384 (PMC3388042; doi:10.1371/journal.pone.0040384)
Supplement: Table S1 — Age based best estimates for non diabetic hypoglycemia in hospitalised patients in non critical care setting. (DOCX) [file pone.0040384.s002.docx]

## Table S1

## Age based best estimates for non diabetic hypoglycemia in hospitalised patients in non critical care setting

| **Cut-off value (mmol/l)** | **2.2** | **2.5** | **2.7** | **3** | **3.3** |
| --- | --- | --- | --- | --- | --- |
| **Age 65 years and over (per 13 494)** | **18** | **20** | **24** | **53** | **74** |
| Lower 95% Confidence Interval Limit | 15 | 18 | 20 | 32 | 43 |
| Upper 95% Confidence Interval Limit | 28 | 31 | 37 | 213 | 201 |
|  |  |  |  |  |  |
| **Incidence (per 10 000)** | **13** | **15** | **18** | **39** | **55** |
| Lower 95% Confidence Interval Limit | 11 | 13 | 15 | 24 | 32 |
| Upper 95% Confidence Interval Limit | 21 | 23 | 27 | 158 | 149 |
|  |  |  |  |  |  |
| **Age below 65 years (per 24 404)** | **15** | **22** | **33** | **65** | **87** |
| Lower 95% Confidence Interval Limit | 14 | 18 | 25 | 45 | 59 |
| Upper 95% Confidence Interval Limit | 22 | 36 | 59 | 115 | 155 |
|  |  |  |  |  |  |
| **Incidence (per 10 000)** | **6** | **9** | **14** | **27** | **36** |
| Lower 95% Confidence Interval Limit | 6 | 7 | 10 | 18 | 24 |
| Upper 95% Confidence Interval Limit | 9 | 15 | 24 | 47 | 64 |
