# Supplementary material for: Hypoglycemia in Non-Diabetic In-Patients: Clinical or Criminal?
Source: PLoS One. 2012 Jul 2;7(7):e40384. doi: 10.1371/journal.pone.0040384 (PMC3388042; doi:10.1371/journal.pone.0040384)
Supplement: Table S2 — Matrix showing co-morbidity linked to hypoglycaemia in patients with glucose <2.7 mmol/l (N = 37). All diagnosis are based on the ICD 10 coding and mostly reflect the codes used in Charlson co-morbidity (Except self harm, dialysis, sepsis and Pneumonia). (DOCX) [file pone.0040384.s003.docx]

## Table S2

Matrix showing co-morbidity linked to hypoglycaemia in patients with glucose <2.7mmol/l (N=37). All diagnosis are based on the ICD 10 coding and mostly reflect the codes used in Charlson co-morbidity (Except self harm, dialysis, sepsis and Pneumonia).

|  | **Renal disease** | **Sepsis** | **Alcohol** | **Cancer** | **Liver disease** | **Pneumonia** | **Congestive heart failure** | **Dementia** | **Self harm** | **Dialysis** | **Occurred on Admission** | **Admitted to investigate hypo** |
| --- | --- | --- | --- | --- | --- | --- | --- | --- | --- | --- | --- | --- |
| **1** | **+** | **.** | **+** | **.** | **+** | **.** | **.** | **.** | **.** | **.** | **.** | **.** |
| **2** | **.** | **.** | **.** | **.** | **.** | **.** | **.** | **.** | **+** | **.** | **+** | **.** |
| **3** | **.** | **.** | **.** | **.** | **.** | **.** | **.** | **.** | **.** | **.** | **.** | **+** |
| **4** | **.** | **.** | **.** | **+** | **.** | **.** | **.** | **.** | **.** | **.** | **.** | **.** |
| **5** | **.** | **.** | **.** | **.** | **.** | **.** | **.** | **.** | **.** | **.** | **+** | **+** |
| **6** | **+** | **+** | **.** | **.** | **.** | **+** | **.** | **.** | **.** | **.** | **+** | **.** |
| **7** | **.** | **.** | **+** | **.** | **+** | **+** | **.** | **.** | **.** | **.** | **.** | **.** |
| **8** | **.** | **.** | **.** | **.** | **.** | **.** | **.** | **.** | **.** | **.** | **+** | **+** |
| **9** | **.** | **.** | **.** | **.** | **.** | **.** | **.** | **.** | **+** | **.** | **+** | **.** |
| **10** | **.** | **.** | **.** | **.** | **.** | **.** | **.** | **.** | **.** | **.** | **+** | **.** |
| **11** | **+** | **+** | **.** | **.** | **.** | **.** | **+** | **.** | **.** | **.** | **.** | **.** |
| **12** | **.** | **.** | **.** | **.** | **.** | **.** | **.** | **.** | **.** | **.** | **+** | **+** |
| **13** | **.** | **+** | **.** | **.** | **.** | **.** | **.** | **.** | **.** | **.** | **+** | **.** |
| **14** | **+** | **+** | **.** | **.** | **.** | **.** | **.** | **.** | **.** | **+** | **.** | **.** |
| **15** | **.** | **.** | **+** | **.** | **.** | **.** | **.** | **.** | **.** | **.** | **+** | **.** |
| **16** | **.** | **.** | **.** | **.** | **.** | **.** | **.** | **+** | **.** | **.** | **.** | **.** |
| **17** | **.** | **+** | **.** | **.** | **.** | **+** | **.** | **.** | **.** | **.** | **.** | **.** |
| **18** | **+** | **.** | **.** | **.** | **.** | **.** | **+** | **.** | **.** | **.** | **+** | **.** |
| **19** | **+** | **.** | **.** | **.** | **+** | **.** | **.** | **.** | **.** | **+** | **.** | **.** |
| **20** | **+** | **+** | **.** | **.** | **.** | **+** | **+** | **.** | **.** | **.** | **.** | **.** |
| **21** | **.** | **.** | **+** | **.** | **.** | **.** | **.** | **.** | **.** | **.** | **+** | **.** |
|  | **Renal disease** | **Sepsis** | **Alcohol** | **Cancer** | **Liver disease** | **Pneumonia** | **Congestive heart failure** | **Dementia** | **Self harm** | **Dialysis** | **Occurred on Admission** | **Admitted to investigate hypo** |
| **22** | **.** | **+** | **.** | **.** | **.** | **+** | **.** | **.** | **.** | **.** | **.** | **.** |
| **23*** | **.** | **.** | **+** | **.** | **+** | **.** | **.** | **.** | **.** | **.** | **.** | **.** |
| **24** | **.** | **.** | **+** | **.** | **.** | **.** | **.** | **.** | **.** | **.** | **+** | **.** |
| **25** | **.** | **+** | **.** | **.** | **.** | **+** | **+** | **.** | **.** | **.** | **.** | **.** |
| **26** | **.** | **+** | **.** | **.** | **.** | **.** | **.** | **.** | **.** | **.** | **.** | **.** |
| **27** | **+** | **.** | **.** | **.** | **.** | **.** | **+** | **.** | **.** | **.** | **+** | **.** |
| **28** | **.** | **.** | **+** | **.** | **+** | **.** | **.** | **.** | **.** | **.** | **+** | **.** |
| **29** | **+** | **+** | **.** | **.** | **.** | **.** | **.** | **.** | **.** | **.** | **.** | **.** |
| **30** | **.** | **.** | **+** | **.** | **.** | **.** | **.** | **.** | **+** | **.** | **+** | **.** |
| **31** | **.** | **.** | **.** | **.** | **.** | **.** | **.** | **.** | **+** | **.** | **+** | **.** |
| **32** | **+** | **.** | **+** | **.** | **.** | **.** | **.** | **.** | **.** | **+** | **.** | **.** |
| **33** | **.** | **.** | **.** | **.** | **.** | **.** | **.** | **.** | **.** | **.** | **+** | **.** |
| **34** | **+** | **.** | **.** | **.** | **.** | **.** | **.** | **.** | **.** | **+** | **.** | **.** |
| **35** | **.** | **+** | **+** | **.** | **+** | **.** | **+** | **.** | **.** | **.** | **.** | **.** |
| **36** | **+** | **.** | **+** | **.** | **.** | **.** | **.** | **.** | **.** | **+** | **.** | **.** |
| **37** | **.** | **.** | **.** | **+** | **.** | **.** | **.** | **.** | **.** | **.** | **.** | **.** |
|  | **12** | **11** | **11** | **2** | **6** | **6** | **6** | **1** | **4** | **5** | **17** | **4** |

* This patient in confused state received 10% dextrose and had an un-recordable 1 blood glucose value (patient with liver disease and alcohol dependence). Documentation of the event was incomplete in the case note review as the notes were deranged. It was included in the analysis as blood glucose value of less than 2.2mmol/l to avoid any underestimation.
