# Supplementary material for: Hypoglycemia in Non-Diabetic In-Patients: Clinical or Criminal?
Source: PLoS One. 2012 Jul 2;7(7):e40384. doi: 10.1371/journal.pone.0040384 (PMC3388042; doi:10.1371/journal.pone.0040384)
Supplement: Text S1 — Three source model layout for estimating the numbers of non-diabetic hypoglycaemia. (DOCX) [file pone.0040384.s004.docx]

## Text S1

**Three source model layout for estimating the numbers of non diabetic hypoglycaemia**

| **Source 1: Anti-hypo treatment** | | | | | | | | |
| --- | --- | --- | --- | --- | --- | --- | --- | --- |
| **Yes No** | | | | | | | | |
|  | |  | **Source 2: Blood Glucose Values** | |  | **Source 2: Blood Glucose Values** | |  |
|  | |  | **Yes** | **No** |  | **Yes** | **No** |  |
|  | | **Yes** | **a** | **b** |  | **e** | **f** |  |
| **Source 3: Discharge Diagnostic code** | |  |  |  |  |  |  |  |
|  |  | **No** | **c** | **d** |  | **g** | **X** |  |
|  | |  |  |  |  |  |  |  |
|  |  |  |  |  |  |  |  |  |

N_obs_ = a+b+c+d+e+f+g (total observed)

N_1_=a+b+c+d (Source 1 total)

N_2_=a+c+e+g (Source 2 total)

N_3_=a+b+e+f (Source 3 total)

X= Unknown value

N_total_=N_obs_ + X

| **Model depicting different interaction between sources** | **DoF** | **Formula to estimate X** |
| --- | --- | --- |
| **Independent** | 3 | X = N_total_ - N_obs_  Where N_total_ is the solution of:  (N_total_-N_1_) (N_total_-N_2_) (N_total_-N_3_) = N_total_^2^(N_total_ - N_obs_ ) |
| **1-2** | 2 | X = (c + d + g)(f) / (a + b + e) |
| **1-3** | 2 | X = (b + d + f)(g) / (a + c + e) |
| **2-3** | 2 | X = (e + f + g)(d) / (a + b + c) |
| **1-2, 1-3** | 1 | X = gf / e |
| **1-2, 2-3** | 1 | X = df / b |
| **1-3, 2-3** | 1 | X = gd / c |
| **1-2, 1-3, 2-3** | 0 | X = (adfg) / (bce) |

Applying correction to the model: For model stability 1 is added to cells b, c and e when performing the analysis

Adapted from: Hook EB, Regal RR. Capture-recapture methods in epidemiology: methods and limitations. [Review] [140 refs][Erratum appears in Am J Epidemiol 1998 Dec 15;148(12):1219]. *Epidemiologic Reviews* 1995; **17**: 243-264.
