# Supplementary material for: Hypoglycemia in Non-Diabetic In-Patients: Clinical or Criminal?
Source: PLoS One. 2012 Jul 2;7(7):e40384. doi: 10.1371/journal.pone.0040384 (PMC3388042; doi:10.1371/journal.pone.0040384)
Supplement: Text S2 — Example: Three source model for estimating the numbers of non diabetic hypoglycaemia (<3.3 mmol/l). (DOCX) [file pone.0040384.s005.docx]

## Text S2

**Example: Three source model for estimating the numbers of non diabetic hypoglycaemia (<3.3mmol/l)**

| **Source 1: Anti-hypo treatment** | | | | | | | | |
| --- | --- | --- | --- | --- | --- | --- | --- | --- |
| **Yes No** | | | | | | | | |
|  | |  | **Source 2: Blood Glucose Values** | |  | **Source 2: Blood Glucose Values** | |  |
|  | |  | **Yes** | **No** |  | **Yes** | **No** |  |
|  | | **Yes** | **1** | **7** |  | **1** | **9** |  |
| **Source 3: Discharge Diagnostic code** | |  |  |  |  |  |  |  |
|  |  | **No** | **5** | **19** |  | **29** | **X** |  |
|  | |  |  |  |  |  |  |  |
|  |  |  |  |  |  |  |  |  |
|  |  |  |  |  |  |  |  |  |

| **Model** | **DoF** | **G^2^** | **P value** | **AIC** | **BIC** | **X** | **N** | **N (lower)** | **N**  **( Upper)** |
| --- | --- | --- | --- | --- | --- | --- | --- | --- | --- |
| **Independent** | 3 | 7.42 | 0.06 | 1.42 | 1.58 | 67 | **141** | 106 | 209 |
| **1-2** | 2 | 5.72 | 0.06 | 1.72 | 1.83 | 44 | **118** | 89 | 187 |
| **1-3** | 2 | 0.77 | 0.68 | -3.23 | -3.12 | 115 | **189** | 124 | 352 |
| **2-3** | 2 | 5.06 | 0.08 | 1.06 | 1.17 | 50 | **124** | 95 | 185 |
| **1-2, 1-3** | 1 | 0.74 | 0.39 | -1.26 | -1.2 | 130 | **204** | 104 | 953 |
| **1-2, 2-3** | 1 | 0.42 | 0.52 | -1.58 | -1.53 | 21 | **95** | 79 | 138 |
| **1-3, 2-3** | 1 | 0.2 | 0.66 | -1.8 | -1.75 | 91 | **165** | 107 | 343 |
| **1-2, 1-3, 2-3** | 0 | 0 | 1 | 0 | 0 | 51 | **125** | 75 | 851 |
